# Hips Do Not Lie: Atypical Pain From Peripartum Pubic Symphysis Diastasis

**DOI:** 10.7759/cureus.71779

**Published:** 2024-10-18

**Authors:** Leonard J Soloniuk, Joshua Lum, Christopher Yeh, Christopher Baker, Ioana F Pasca

**Affiliations:** 1 Department of Anesthesiology, Loma Linda University Medical Center, Loma Linda, USA; 2 Department of Anesthesiology and Perioperative Medicine, Riverside University Health System Medical Center, Moreno Valley, USA; 3 Department of Gynecology and Obstetrics, Loma Linda University Medical Center, Loma Linda, USA; 4 School of Medicine, Loma Linda University School of Medicine, Loma Linda, USA; 5 Osteopathic Medicine, Philadelphia College of Osteopathic Medicine, Moultrie, USA; 6 School of Medicine, University of California Riverside School of Medicine, Riverside, USA

**Keywords:** labour epidural analgesia, nerve paresthesia, nulliparous, obstetric anesthesia, pubic symphysis diastasis

## Abstract

Pubic symphysis diastasis (PSD) is the widening of the pubic symphysis which can occur during the peripartum period. PSD commonly presents as pelvic pain with associated neuropathies rarely reported. In this report we describe the unique presentation of PSD with associated motor and sensory deficits in a 19-year-old postpartum patient. Two days following vaginal delivery, the patient complained of diffuse body pain, generalized weakness, and lower extremity paresthesia without any other neurological deficits. PSD was diagnosed by radiograph of the pelvis and her symptoms swiftly improved following conservative management of the diastasis. Further, we discuss the potential etiology of this patient’s presentation as well as differential diagnosis with similar presentations.

## Introduction

Changes occurring throughout gestation results in dramatic alterations to maternal anatomy and physiology, including the pubic symphysis. The pubic symphysis is a non-vascular joint supported by four ligaments and the muscles of the pelvic floor comprising the anterior border of the pelvic inlet and outlet [1]. Under normal circumstances, the pubic symphysis serves to absorb shock from the pelvic ring during ambulation and stabilizes the joint when bending, standing and during one-legged stance [1]. Relaxation of the pubic symphysis occurs during the first trimester of pregnancy due to hormonal changes and during child birth, with up to three to five mm dilation of the pubic symphysis being physiologically normal [1]. Laxity of the pelvic ligaments spanning the sacroiliac joints and pubic symphysis allows for a small increase in the size of the pelvic inlet [2].

Pubic symphysis diastasis (PSD), defined as excessive widening of the pubic symphysis of one cm or greater, is a relatively rare complication of vaginal delivery first reported in 1690 [3], with an incidence ranging from 1:300 to 1:30,000 [1]. The normal radiographic separation of the pubic symphysis is a width of four to five mm [4]. Risk factors for developing PSD include fetal macrosomia, nulliparity, precipitous labor, narrow pelvic outlet, cephalo-pelvic disproportion, epidural anesthesia, and previous pelvic trauma [4,5]. A classic presentation of PSD includes symptoms such as lower abdominal pain and anterior pelvic pain with radiation down the legs [5]. Additionally, patients can experience difficulty with weight-bearing, bending forward, and ambulating secondary to pain, as well as low back pain and pelvic joint pain, exacerbated by sacroiliac provocation tests. It has been suggested that the combination of these postpartum symptoms be termed pelvic joint syndrome [6].

Associated neuropathies are rare but have been reported, although the etiology often remains uncertain. The severity of symptoms has not been correlated with the degree of diastasis. Consideration of PSD, particularly with atypical presentation, can lead to its prompt recognition, potentially avoiding the overuse of resources and providing proper management in a timely manner. In this report, we describe the unique presentation of PSD with associated motor and sensory deficits in a 19-year-old postpartum patient.

Express Health Insurance Portability and Accountability Act (HIPAA) authorization for publication was obtained from the patient, ensuring compliance with the applicable CAse REports (CARE) guidelines under Enhancing the Quality and Transparency of Health Research (EQUATOR) framework for case reports.

## Case presentation

A 19-year-old female gravida 1 para 0, at 40 weeks 1 day gestation, underwent induction of labor for spontaneous rupture of membranes. Seven hours after induction, the patient requested epidural analgesia, which was administered without complication. Excellent pain control was maintained throughout the delivery. Excessive hip flexion or the McRoberts maneuver was not utilized. The second stage of labor lasted for 48 minutes. There was vaginal delivery of a healthy male infant weighing 3330 grams. The delivery was complicated by postpartum hemorrhage treated with IV oxytocin and placement of an intrauterine vacuum-induced hemorrhage control device. Two days following the vaginal delivery, the patient complained of diffuse body pain rated at a severity of 10/10, generalized weakness, and lower extremity paresthesia. She was unable to ambulate due to pain and weakness.

**Figure 1:**
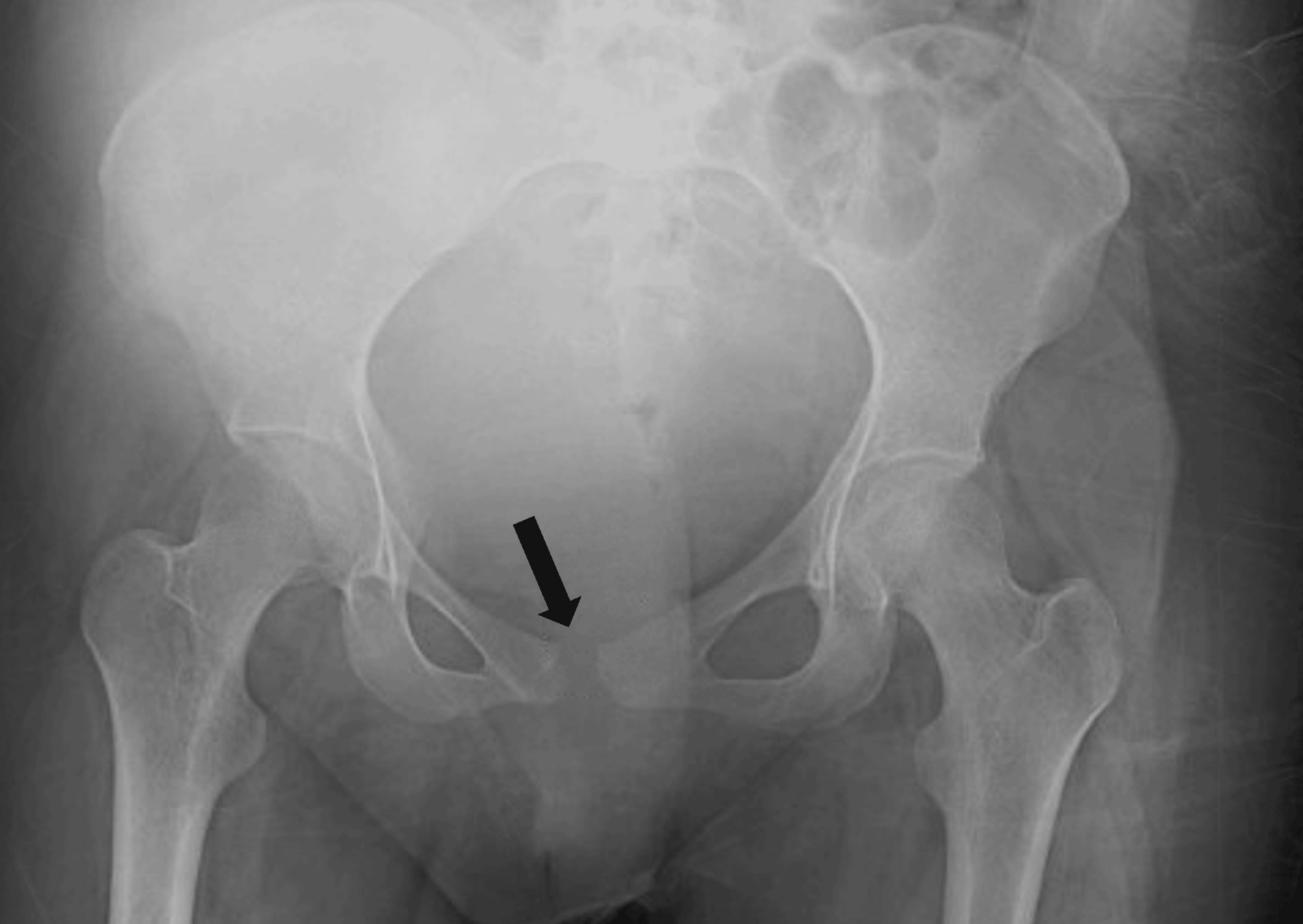
AP pelvis radiograph with black arrow indicating a pubic symphysis diastasis of 1.04 cm Anteroposterior: AP

A pain diagram indicated that the most severe pain was located anteriorly in a band stretching across the suprapubic area between the anterior-inferior iliac spines, and posteriorly across the L5 vertebral level. The patient denied any saddle anesthesia or changes in bowel and bladder function. Physical examination revealed a distraught woman with very guarded movements. There was diffuse tenderness over the lumbosacral spine, abdomen, and pelvis with diffuse bilateral distal lower extremity numbness to light touch. The motor exam showed reduced 4/5 muscle strength for hip flexion and knee extension bilaterally. Achilles and patellar reflexes were brisk bilaterally. Magnetic resonance imaging (MRI) of the thoracic and lumbar spine two days postpartum showed no abnormalities on review by a radiologist and one of the authors. Orthopedic spine surgery and neurology were consulted and had no further recommendations. The psychiatric evaluation reported that the patient did not meet any criteria for an anxiety disorder, depression, psychosis, or post-traumatic stress disorder. The patient was placed on a multimodal pain regimen including acetaminophen, duloxetine, gabapentin, methocarbamol, and ibuprofen, with only modest improvement in her pain and function. The following day an anteroposterior (AP) pelvic radiograph was performed, revealing a widened pubic symphysis of 1.04 cm (Figure 1).

PSD was diagnosed three days postpartum, and the patient was treated with an external compression belt and physical therapy. The patient had rapid improvement in her pain and function with resolution of her motor and sensory deficits. The patient was safely discharged home on postpartum day six. At her four-week postpartum follow-up, the patient was recovering as expected without any pelvic pain or neurologic deficits. 

## Discussion

Here, we present an atypical presentation of peripartum PSD. Rather than traditional pelvic girdle pain, the patient presented with diffuse total body pain, paresthesia, and bilateral lower extremity weakness on exam. The patient had several risk factors for PSD, including nulliparity and the use of epidural analgesia.

Literature review revealed one similar case report describing a 31-year-old female, gravida 3 para 2, at 41 weeks 6 days of gestation [7]. McRobert’s maneuver was utilized and the patient vaginally delivered a 4314 grams infant. After delivery, she immediately experienced bilateral lower extremity weakness and paresthesia. Radiographs confirmed a PSD with 5 cm separation and inferior displacement. Her symptoms resolved within two days with conservative management. The authors suggest the patient developed a femoral nerve palsy from the marked flexion and abduction at the hips during the McRobert’s maneuver. Lower extremity nerve injury associated with childbirth is estimated to occur with an incidence of 0.3% to 2.3% [8]. While there are similarities between the case reported by Gherman et al. and the current case, neither femoral nerve palsy nor other peripheral nerve palsies common in parturients adequately explain our patient’s clinical findings [7,9].

Common nerves that can be affected in parturients include the lumbosacral plexus and common peroneal nerve, leading to footdrop and numbness to the dorsum of the foot and lateral aspect of the lower leg. Also possibly affected are the lateral femoral cutaneous, femoral, and obturator nerves, which often occur concurrently, causing numbness over the thighs and weakness in hip flexion, knee extension, and hip adduction [9]. The pubic symphysis reportedly receives innervation from several nerves, including the pudendal, genitofemoral, iliohypogastric, and ilioinguinal nerves [10]. However, referred pain or palsies associated with these nerves fail to completely describe the neurologic symptoms experienced in the current case. 

Several anesthesia-related complications with a similar presentation to the present case include arachnoiditis, epidural hematomas, epidural abscess, direct nerve root injuries, and spinal cord trauma. The clinical presentation of arachnoiditis secondary to epidural analgesia has been described to include difficulty walking, weakness and decreased sensation in lower extremities [11]. The prevalence of arachnoiditis is difficult to predict due to its rarity, with most of the literature on the topic consisting of case reports. Epidural hematomas can present with back pain (radicular), bladder dysfunction, sensory, and motor deficits with varying incidences reported in literature from 1:26,400 [12], to 21:1.7 million [13], following epidural anesthesia. Epidural abscess is a rare but serious complication of epidural analgesia with varying incidences reported in literature of 1:200,000 to 1:500,000 [14], and 12:1.7 million [13], following epidural catheter placements. An epidural abscess can present with fever, back tenderness, headache, malaise, nerve root pain, motor deficits, and sensory deficits [9]. Traumatic cord lesions are a rare complication of neuraxial anesthesia, with an estimated incidence of 8:1.7 million epidural catheter placements [13]. However, an unremarkable MRI of the thoracic and lumbar spine in this patient makes the above anesthesia-related neurological complications unlikely.

There is growing awareness of the rare condition known as hereditary neuropathy, which has an increased liability to pressure palsy (HNPP). Individuals with HNPP experience recurrent motor and sensory neuropathies following brief nerve compression [15]. It is an autosomal dominant disorder that develops in childhood and can go unnoticed due to mild forms being mistaken for peripheral neuropathies or peripheral nerve compression, such as carpal tunnel syndrome. However, the patient does not have a family or medical history of peripheral nerve compression syndromes or recurrent neuropathies, making this diagnosis less likely.

Initial treatment for PSD is conservative management with mechanical stabilization of the pelvis with an external compression belt along with pelvic floor strengthening exercises. Other non-invasive interventions include cryotherapy, nonsteroidal anti-inflammatory drugs (NSAIDs), and possibly glucocorticoids. If symptoms persist despite conservative measures or the patient has a diastasis greater than four cm, elective surgery is considered [1,4,16]. For patients with associated neurologic deficits, clinical suspicion for alternative and reversible causes should remain high. The choice of management as it relates to the current patient included first ruling out reversible causes for her neurologic deficits with an MRI of the thoracic and lumbar spine in addition to consulting neurology. With an unremarkable MRI and CT, as well as identification of PSD less than four cm on the pelvic radiograph, the decision to pursue conservative management was made. 

## Conclusions

In conclusion, the course of her clinical presentation and subsequent swift resolution of symptoms in response to standard therapy for PSD makes it likely that the sensory and motor manifestations were secondary to a complex syndrome that included PSD. Although her symptoms could potentially be attributed to transient nerve palsies, including the femoral nerve and lumbosacral plexus, initially masked by the epidural analgesia, this does not explain the rapid improvement after receiving appropriate treatment for PSD. This case underlines how clinicians should consider the differential of PSD in postpartum patients experiencing pain and neurologic symptoms, and supports a broader clinical awareness of atypical PSD symptoms which can be successfully managed conservatively in the absence of a large PSD (>4cm) or other etiologies of neurological deficits requiring surgical intervention. 
